# Small bowel perforation and death caused by anaplastic thyroid carcinoma metastasis in a patient with concomitant colonic and bilateral breast carcinoma.

**DOI:** 10.4322/acr.2021.255

**Published:** 2021-03-29

**Authors:** Jan Hrudka, Ivana Švadlenková

**Affiliations:** 1 Charles University, 3rd Faculty of Medicine, University Hospital Královské Vinohrady, Department of Pathology, Prague, Czech Republic; 2 Charles University, 3rd Faculty of Medicine, University Hospital Královské Vinohrady, Department of General Surgery, Prague, Czech Republic

**Keywords:** Carcinoma, Intestinal Perforation, Neoplasm Metastasis, Thyroid Carcinoma, Anaplastic

## Abstract

Undifferentiated or anaplastic thyroid carcinoma (ATC) is rare and one of the most aggressive human malignancies. The tumor is usually voluminous and fast-growing and mostly affects older women. The most common sites of distant metastases are the lungs, brain, and bones. Herein, we describe the case of a 66-year-old woman with a history of bilateral breast carcinoma and ATC, who presented with an acute abdomen and subsequently died. At autopsy, an isolated metastasis of ATC in the small intestine leading to bowel perforation was found. Moreover, there was adenocarcinoma in the descending colon. The review of extra-abdominal malignancies metastasizing to bowel and coincidence of breast and thyroid carcinoma is included.

## INTRODUCTION

Undifferentiated or anaplastic thyroid carcinoma (ATC) is rare and is one of the most aggressive human malignancies. The ATC is composed of undifferentiated thyroid follicular cells. It occurs typically in older females. The majority of patients succumb to the metastatic disease within 6 months. The most common sites of metastases are the lungs, brain, and bones.^1^ Herein, we describe an unusual case of ATC metastasizing to the small bowel without evidence of any other distant metastasis, resulting in the bowel perforation and peritonitis, which subsequently caused the patient's death.

## CASE REPORT

**Figure 1:**
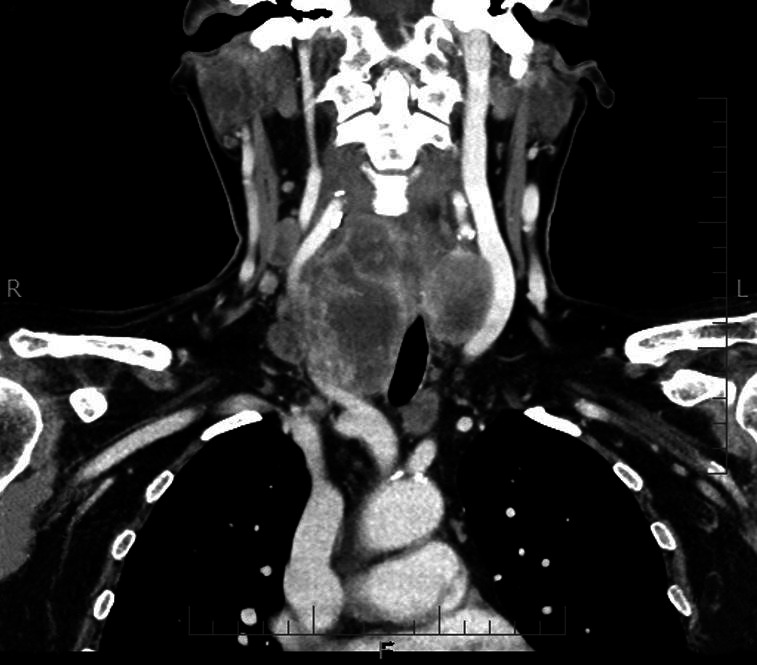
Cervical computed tomography scan showing voluminous tumor of the thyroid gland with a preponderating mass in the right lobe embedding the right carotid artery.

A 66-year-old Caucasian woman presented to the Surgery Department with acute abdominal signs, following a 2-day history of fever and abdominal pain. At the presentation, the patient was febrile (38.1°C), with notable abdominal distension, without nausea. There were palpation signs of peritonism, with bilateral hypogastric pain. Intestinal sounds were present. Her medical history included type-2 diabetes, bilateral breast cancer, and ATC. The breast cancers were both pT1N0 invasive ductal breast carcinoma, operated on 2 and 5 years ago. After the first mastectomy, the patient was also submitted to radiotherapy, and after the second surgery, she was kept on tamoxifen. An ATC was diagnosed 2 months ago. She was submitted to a palliative right side hemithyroidectomy and right block neck dissection of groups II–V because of a voluminous bilateral tumor of the thyroid involving the carotid artery, and four out of six resected lymph nodes (Figure 1).

The laboratory workup revealed a marked elevation of C-reactive protein (167.8 mg/L; reference range [RR] <5) and leukocytosis 21.9 × 10^9^/l (RR 4.0-10.0). The abdominal computer tomography revealed pneumoperitoneum. An exploratory laparotomy was scheduled, but while waiting for this, the patient developed shortness of breath, a cough, inspiratory crackles, and room-air pulse oximetry of 70%. The patient did not report any chest pain. Due to the resulting loss of consciousness, she underwent orotracheal intubation but thereafter experienced a cardiac arrest. Cardiopulmonary resuscitation maneuvers were performed; however, the patient did not survive. Aiming to clarify the cause of death, an autopsy was performed.

## AUTOPSY FINDINGS

**Figure 2:**
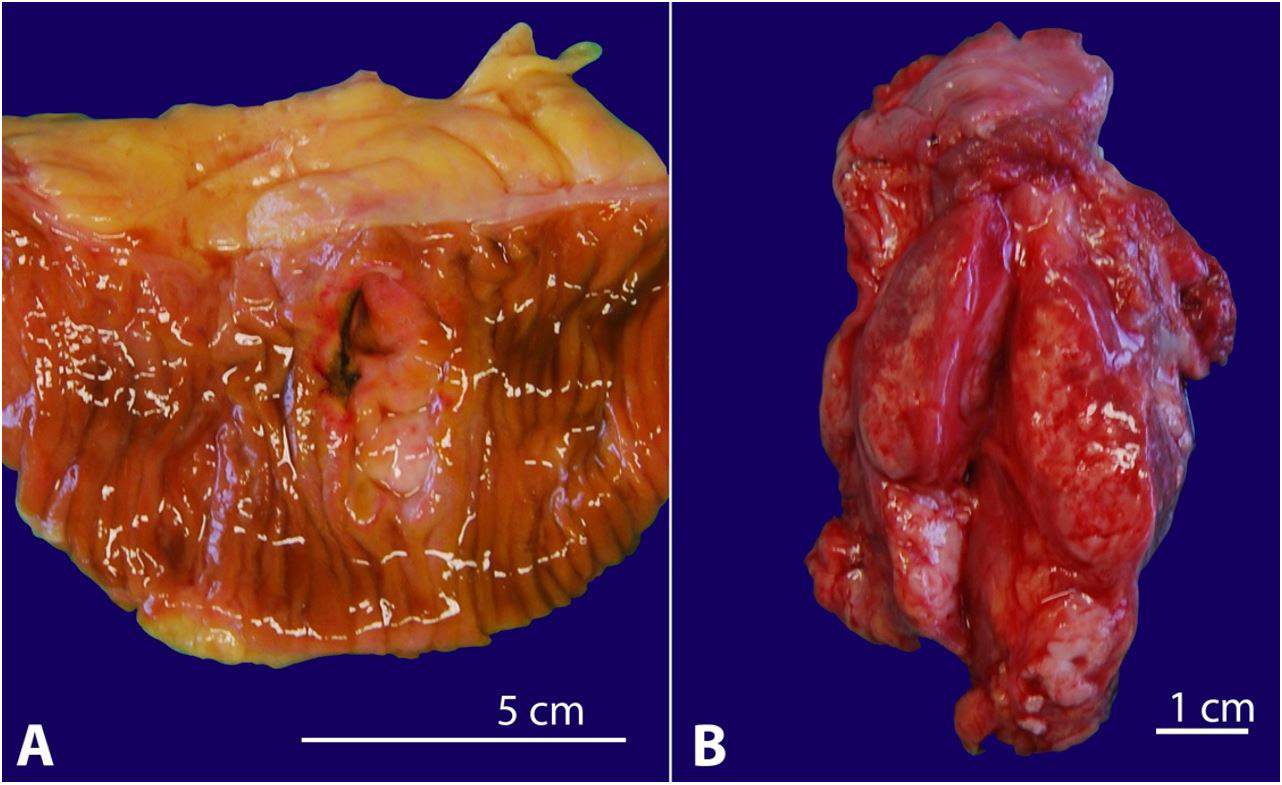
A – Gross view of a small bowel segment showing a perforated ulceration measuring approx. 30 × 20 mm. B – Gross picture of the left lobe of the thyroid gland containing a white, firm, tumorous mass with the adjacent lymph node infiltrated by the tumor.

The external examination revealed a scar in the patient's neck region, older scars related to the bilateral mastectomy, and a bloated abdomen. The abdominal cavity opening revealed a markedly distended intestine, and the visceral peritoneum was covered with pus. There was a perforation of the small intestine measuring 4 mm at 2 meters from the ileocecal junction. An indurated ulceration with an elevated firm margin measuring 30 × 20 mm underlying the perforation was found in the intestinal mucosa (Figure 2A). Also, there was a cauliflower-shaped polypoid tumor in the mucosa of the descending colon.

The right thyroid lobe was absent, and the remaining left lobe was markedly enlarged and firm. Almost the entire bulk of the gland tissue was replaced by a whitish and partly necrotic tumor mass measuring 70 × 30 × 10 mm (Figure 2B), which infiltrated the surrounding tissue and lymph nodes.

The lungs were heavy and boggy. The right lung weighed 940 g (mean RR: 450 g), and the left lung weighed 860 g (mean RR: 375 g). A significant amount of foamy fluid drained out from the cut section of the lungs, consistent with lung edema. No metastatic foci were found in the lung parenchyma. The heart weighed 460 g (mean RR: 280 g) and showed left ventricular hypertrophy. Advanced calcifying atherosclerosis was found in the aorta and major arteries.

A pendulating polyp in the uterine cavity was incidentally found. Besides the tumors in the thyroid and adjacent lymph nodes, and in the small intestine and colon, there were no other sites of metastases.

**Figure 3:**
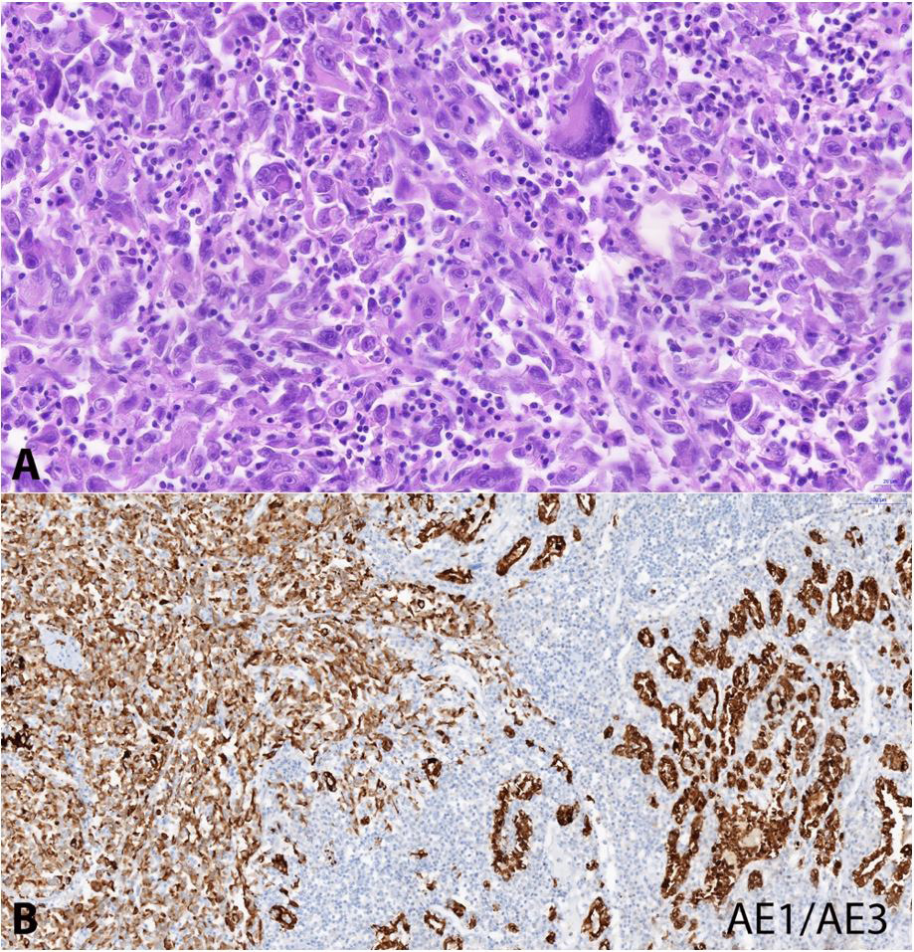
Photomicrograph of the thyroid tumor. A – A malignant tumor consisting of highly pleomorphic epithelioid cells with marked cytonuclear atypia, including bizarre cells and mitotic figures (H&E, 44.8X). B – Cytokeratin positivity in the tumor cells (left) and the non-neoplastic thyroid follicles (right) (AE1/3 15,8x).

The histology of the thyroid gland tumor consisted of pleomorphic epithelioid cells with marked cytonuclear atypia, rich mitotic activity, and admixed chronic inflammatory cells and necrosis (Figure 3A). This finding was similar to the hemithyroidectomy specimen histological report of the previous 2 months; that is, undifferentiated (anaplastic) thyroid carcinoma. In the vicinity of the thyroid gland, two lymph nodes with metastases were found. Immunohistochemically, the tumor was positive for cytokeratin AE1/3 (Figure 3B), whereas the remaining examined markers, paired box 8 (PAX8), thyroid transcription factor-1 (TTF-1), chromogranin A, carcinoembryonic antigen (CEA), and epithelial membrane antigen (EMA), were negative. Non-neoplastic thyroid follicular cells showed strong nuclear TTF-1-positivity. Expression of p53 was wild type in the tumor. The proliferation activity (Ki67) was present in approximately 80% of tumor cell nuclei.

**Figure 4:**
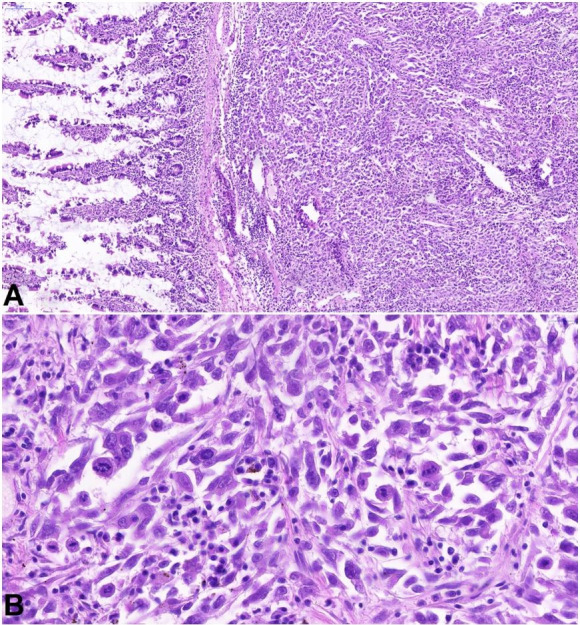
Photomicrograph of the intestinal perforation site showing a pleomorphic tumor with similar morphology to the thyroid carcinoma infiltrating the small bowel submucosa (A), marked cytonuclear atypia, and mitotic activity in detail (B). (H&E, A 11.7X, B 57,0X.).

A similar malignant epithelioid tumor was found in the small intestine wall at the perforated ulceration site (Figure 4). We considered this lesion as a perforated metastasis of ATC in the small intestine. The tumor cells were cytokeratin 20- and CDX2-negative. The p53 expression was wild type. Fibrin precipitations and abundant neutrophils were present on the visceral peritoneum, consistent with the diagnosis of purulent peritonitis.

**Figure 5:**
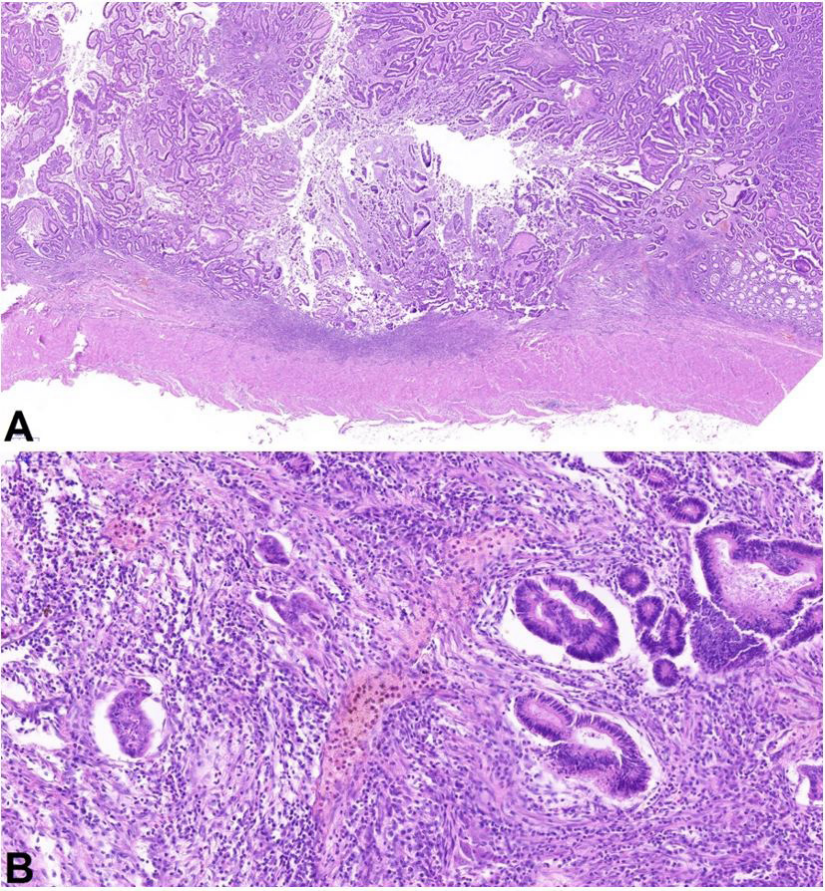
Photomicrograph of the intestinal adenocarcinoma of the descending colon, with submucosal invasion, originating in a tubular adenoma. (H&E, A 2.1X, B 21,3X.).

A well-differentiated, invasive, intestinal adenocarcinoma with submucosal invasion originating in a tubular adenoma was found in the descending colon without metastases on a thorough gross examination (Figure 5).

There was a corporal endometrial polyp in the uterine cavity. The histology of the left ventricular myocardium displayed hypertrophy and small dispersed scars. Alveolar edema was found in both lungs, without hyaline membranes.

The autopsy findings rendered the diagnosis of an ATC as the underlying disease, complicated by an isolated perforated metastasis in the small intestine. Purulent peritonitis and sepsis were the immediate cause of death. With the patient's history of metachronous bilateral breast cancer, and an incidental finding of colonic adenocarcinoma, the patient suffered from tumor quadruplicity.

## DISCUSSION

ATC is a highly aggressive tumor composed of undifferentiated cells, which may arise de novo or in a differentiated thyroid malignancy; that is, papillary carcinoma. ATC arising in a papillary thyroid carcinoma has been described in relation to the expression of cancer stem cell markers. These have been documented in ATCs as a hallmark of adverse outcome.^2^

Grossly, the ATCs are usually voluminous and infiltrative and are light and fleshy on the cut section—often with necrosis and hemorrhage. Microscopically, the tumor cells are often pleomorphic with significant cytonuclear atypia. A sarcomatoid, giant cell or epithelioid pattern may be observed. Vascular invasion is a frequent feature.^1^

Histopathological diagnosis may be challenging. PAX8 is a transcription factor expressed in normal and neoplastic thyroid follicular epithelium widely used by pathologists as an immunohistochemical marker of thyroid origin in neoplasms. However, the sensitivity is notably lower in ATC than that believed in previous studies,^3^ whereas a recent study by Lai et al.^4^ proved a PAX8-positivity in only 54% of ATCs. Even the cytokeratins and EMA documenting their epithelial nature are expressed in only 55%–65% of ATCs.^5^ ^,^ ^6^ A mutation of tumor suppressor gene TP53 occurs in most ATCs^7^ ^,^ ^8^ and may be proven by strong diffuse positivity or full negativity of p53 immunohistochemistry. However, this is unspecific; in our case, there was a p53 wild type expression. In regard to differential diagnosis, poorly differentiated thyroid carcinoma as an unusual variant of papillary carcinoma may be considered due to abundant mitotic figures and necrosis^9^ observed in our case. Moreover, there is an exceedingly rare spindle cell variant of papillary carcinoma distinct from ATC described in the literature.^10^ Nevertheless, bizarre high-grade nuclear atypia, PAX8- and TTF-1-negativity support rather our diagnosis of ATC. Spindle-shaped cells may occur in medullary thyroid carcinoma, which may be dominated or exclusively composed of spindle cells.^9^ However, we prefer ATC diagnosis because of chromogranin-negativity and marked cytonuclear pleomorphism. Sarcomas of the thyroid gland, both primary and metastatic, are extraordinarily rare, and we may exclude these by the strong cytokeratin positivity in our case.

There are ATC cases that lack a reliable diagnostic marker. Distinguishing the primary thyroid tumor from metastasis often remains a diagnosis of exclusion, which was evidenced by the autopsy in our case.

The ATC annual incidence counts for one to two cases per million persons.^11^ The prognosis is extremely poor, with a median 1-year survival rate of only 10%–20%.^12^ ^-^ ^14^ The tumor occurs in older persons and is slightly more frequent in women. The patients often present with metastatic involvement of regional lymph nodes at the diagnosis. Of the patients, 30%–40% have distant metastases—most often involving the lung, bone, and brain.^1^ Metastatic involvement of the intestine is unusual.

Generally, symptomatic metastases of extra-abdominal malignancies in the intestines are rare. Regarding small bowel metastasis of thyroid carcinoma, we found five references in the medical literature^15^ ^-^ ^19^ —all are related to ATC, three of them presented with intestinal perforation,^17^ ^-^ ^19^ as in our case. The most common primary tumors metastasizing to the small bowel are lung carcinomas^20^ ^-^ ^42^ and malignant melanoma,^43^ ^-^ ^48^ followed by renal cell carcinoma.^49^ ^,^ ^50^ Rare cases of testicular seminoma,^51^ osteosarcoma,^52^ ^,^ ^53^ and Ewing sarcoma^54^ have also been described. Symptoms of intestinal metastasis described in the literature include intussusception,^16^ ^,^ ^20^ ^-^ ^29^ ^,^ ^43^ ^-^ ^54^ and perforation^17^ ^-^ ^19^ ^,^ ^30^ ^-^ ^42^.

The curative treatment of ATC is multimodal and consists of surgery and external beam radiation therapy with radio-sensitizing chemotherapy.^11^ The combined therapy tends to have a better outcome than a single modality alone.^55^ In terms of targeted therapy, there are some successful trials with selective BRAF-inhibitors, whereas BRAF mutation is seen in 25% of tumors.^11^ ^,^ ^56^ ^-^ ^58^

There is an unsolved question of multiple malignancies (bilateral breast carcinoma, colonic carcinoma, thyroid carcinoma) in our patient. Notwithstanding a possibility of hereditary syndromes, there is evidence suggesting that breast cancer and thyroid cancer occur together in the same female patients more frequently than would be expected by chance, synchronously or metachronously.^59^ ^,^ ^60^ There are several explanations being subject of recent research, focusing i.e., on detection bias, treatment effect, iodine intake, folate metabolism, obesity, gonadal hormones, thyroid hormone, and genetic susceptibility.^61^ ^,^ ^62^ Based on endocrinological studies, iodine deficiency may stimulate the gonadotropin secretion and then result in a hyperestrogenic state, which increases the risk of breast carcinoma.^63^ Because thyroid carcinoma is highly prevalent among fertile women, hormonal and reproductive factors may also be involved in its incidence.^64^ Since these references mostly describe papillary thyroid carcinoma, we cannot exclude this as a lesion preexistent to ATC in our patient. Moreover, thyroid and breast carcinoma may be induced by radioactive iodine administration or by external beam radiation to the breast, respectively. Traditional risk factors, obesity, and radiation exposure increase the risk of both thyroid and breast cancer.^62^ ^,^ ^65^ ^,^ ^66^ In regard to genetic susceptibility, Goldgar et al.^67^ observed excess thyroid cancer rates among first-degree relatives of probands with breast cancer and vice versa. Cowden syndrome is an example of a hereditary tumor syndrome known to increase the risk of developing both breast and differentiated thyroid cancer in the same individual^68^ as no skin or gastrointestinal hamartomas were indicating Cowden syndrome in our case.

In conclusion, we report the unique case of a female patient suffering from tumor quadruplicity, while an ATC was the lethal tumor metastasizing to the small intestine leading to intestinal perforation, purulent peritonitis, and the patient's death. Due to the autopsy nature of our material, the question of a hereditary tumor syndrome remains unresolved. We hypothesize that the presence of the ATC was induced by breast cancer radiotherapy many years ago. The patient's general practitioner was informed of the autopsy results and all the facts noted above, with a recommendation to conduct a genetic examination of the patient's family.
